# Pharmacokinetic-pharmacodynamic relationship of anesthetic drugs: from modeling to clinical use

**DOI:** 10.12688/f1000research.6601.1

**Published:** 2015-11-18

**Authors:** Valerie Billard

**Affiliations:** 1 Department of Anesthesia and surgical intensive care, Gustave Roussy Cancer Center, 114, rue Édouard-Vaillant, VILLEJUIF, 94805, France

**Keywords:** pharmacokinetic, pharmacodynamic, anesthetic, Anesthesia, anesthetic drugs

## Abstract

Anesthesia is a combination of unconsciousness, amnesia, and analgesia, expressed in sleeping patients by limited reaction to noxious stimulations. It is achieved by several classes of drugs, acting mainly on central nervous system. Compared to other therapeutic families, the anesthetic drugs, administered by intravenous or pulmonary route, are quickly distributed in the blood and induce in a few minutes effects that are fully reversible within minutes or hours. These effects change in parallel with the concentration of the drug, and the concentration time course of the drug follows with a reasonable precision mathematical models based on the Fick principle.

Therefore, understanding concentration time course allows adjusting the dosing delivery scheme in order to control the effects.

The purpose of this short review is to describe the basis of pharmacokinetics and modeling, the concentration-effects relationship, and drug interactions modeling to offer to anesthesiologists and non-anesthesiologists an overview of the rules to follow to optimize anesthetic drug delivery.

## Introduction

Anesthesia is a complex state including several reversible therapeutic effects such as loss of consciousness and recall or lack of response to variable noxious stimulations coming from surgery or anesthetic management. Some effects are quantitative, such as electroencephalogram (EEG) or blood pressure changes, and the intensity of effects increases with the dose. Others are quantal (yes or no), such as being asleep or the absence of movement response to surgical incision. The probability of these quantal effects increases with the dose.

This is also true for adverse effects (such as hypotension, bradycardia, and respiratory depression), although they usually occur at higher doses than therapeutic effects. Therefore, drug dosages should be chosen to maintain the patient inside a therapeutic window, and dosing should be large enough to achieve therapeutic effects but small enough to avoid late recovery or adverse effects. The wider the therapeutic window, the safer the drug, but even for modern anesthetic drugs, this therapeutic window may be narrow in some patients, depending on age, physiological status, or drug combinations. Anesthetists should first target a window which they consider likely to be adequate for the patient and the procedure and then assess whether the anesthetic effect is at the level expected and titrate if this is not the case.

Acting on the central nervous system (CNS), which is a fatty structure, all anesthetic drugs are lipophilic. Consequently, every single dose administered follows a distribution process in the body, and the fraction of dose reaching the CNS competes with the biggest inactive fraction distributed in the blood, muscles, and fat at different rates. At the same time, the last fraction of the dose disappears irreversibly from the body through metabolism or excretion. For anesthetic procedures, lasting from a few minutes to a few hours, steady state is never reached. Consequently, the effects related to a dose will change over time because of this balance but, for most drugs, will be parallel to the concentration at the site of effect.

Understanding the pharmacokinetics (PK) allows clinicians to adjust the delivery scheme in order to control the concentration at the site of effect in the present and in the future (control of recovery). Understanding pharmacodynamics (PD) (that is, the relationship between concentration and intensity of effects) helps in titrating anesthesia delivery according to individual needs and to successive surgical end-points.

The aim of this overview is to present the concepts describing this pharmacokinetic-pharmacodynamic (PKPD) relationship and their clinical implications. Although the PKPD relationship has also been described for volatile or local anesthetics in regional anesthesia, we will focus here on intravenous drugs, except for drug interactions.

Many scientists have helped describe and validate these concepts, as shown in the references. In this short review, I may have forgotten some important names or keystone articles. I hope the authors will forgive me and will recognize their discoveries in the full picture.

## Pharmacokinetics and modeling: compartmental models

**Figure 1.**
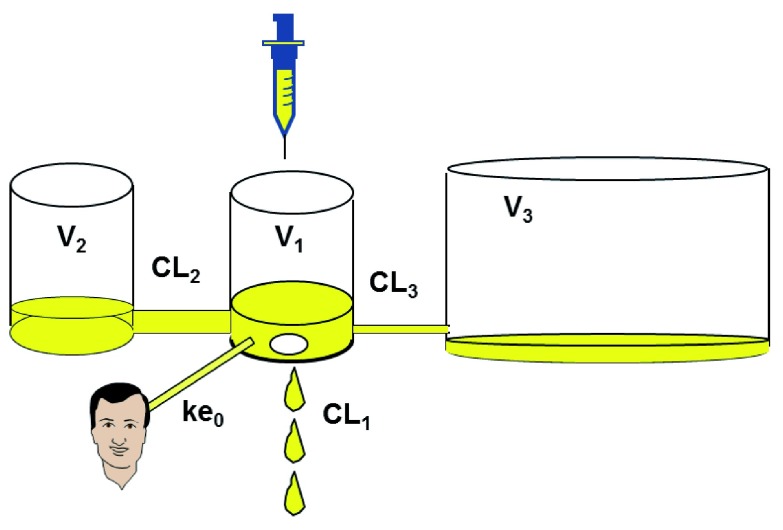
Three-compartment pharmacokinetic model. CL _1_ = k _10_*V _1_, CL _2_ = k _12_*V _1_, CL _3_ = k _13_*V _1_, V _2_ = CL _2_/k _21_, V _3_ = CL _3_/k _31_. CL: clearance; k: micro-constant; V: distribution volume.

After giving a dose of an anesthetic drug (bolus or infusion) and drawing blood assays, scientists have observed that the time course of plasma concentration may be modeled by using a two- or three-compartment model with an acceptable precision ^1^. This model can be fully characterized by three volumes of distribution, two distribution clearances, and one elimination clearance (or a bunch of six micro-constants describing exchanges; Figure 1). It is specific to the drug, independent of the dose, and linear: doubling the dose will double the plasma concentration at any time, and if two doses are given (for example, two boluses or bolus + infusion), the plasma concentration will be the sum of the concentrations resulting from each dose. This is called the “superposition principle”.

PK models describe the time course of plasma concentration, estimated by using the drug doses delivered over time and patient characteristics such as weight, height, age, and gender. But the time course of effects is always delayed and attenuated compared with that of plasma concentration because the site of effect is not plasma but CNS (or muscles for muscle relaxants) ^2^. To model the time course of effect, Sheiner and colleagues added the effect compartment as a fourth compartment to the PK model, having a negligible volume and assumed to be synchronized with effects ^3^. The relationship between plasma and effect-site concentration may be described by a single time constant called ke0, which extends the PK to a PKPD model. At steady state, plasma and effect-site concentration are identical ( Figure 1).

Whereas early studies determined PK parameters separately for every patient and averaged them in a second step (which required many samples per patient during and after administration), most of the modeling since the 1990s used population analysis with mixed-effects modeling ^4^. In this approach, samples from all patients are considered together. The modeling provides not only the typical values of the parameters but also an estimation of inter-individual and intra-individual variability of parameters and predicted concentration. Using all samples in the same fit requires a lower number of samples per patient.

Moreover, this type of modeling allows clinicians to add physiological covariates to the model (such as weight, lean body mass [LBM], and age, as shown in Table 1 ^5– 7^), added as additive or scaling factors ^8^, which may improve the fit and thus the prediction of the plasma or effect-site concentration.

**Table 1.** Some of the numerous models published for propofol.

Parameters	Marsh *et al*. ^5^	Schnider *et al*. ^6^	Coppens *et al*. ^7^
V _1_, L	0.228 * W	4.27	0.4584 * W
V _2_, L	0.4643 * W	18.9 − 0.391 * (age − 53)	0.950 * W
V _3_, L	2.895 * W	238	5.820 * W
CL _1_, L/min	0.027 * W	1.89 + (W − 77) * 0.0456 − (LBM − 59) * 0.0681 + (H − 177) * 0.0264	0.0699 * W * W ^−0.3^
CL _2_, L/min	0.0255 * W	1.29 − 0.024 * (age - 53)	0.0522 * W
CL _3_, L/min	0.0095 * W	0.836	0.0192 * W

CL: clearance; H: height; LBM: lean body mass; V: volume; W: weight.

### Clinical use of pharmacokinetic-pharmacodynamic models

For all anesthetic drugs, one or several PKPD compartmental models have been published, most of them using a quantitative effect such as EEG to determine ke0. The models are implemented in simulation software available on the web:

-      Stanpump, Stelpump, or Ivasim: http://www.opentci.org
-      Rugloop: http://www.demed.be/rugloop.htm
-      Pkpdtools: http://www.pkpdtools.com
-      Tivatrainer: http://www.eurosiva.org/TivaTrainer/tivatrainer_main.htm

After the drug, the patient characteristics, and the PK model are chosen, these software packages simulate the time course of plasma and effect-site concentration for any drug-delivery scheme. Some of them, such as Stanpump or Rugloop, can drive a syringe pump, combining the delivery data setup by the user and the events coming from the patient (such as venous line occlusion and empty syringe) to display past, present, and future predicted concentration. Their clinical use should be limited to research study with institutional review board approval and insurance, since none of the software packages mentioned above has a CE (“European Conformity”) mark or US Food and Drug Administration (FDA) approval for clinical use.

By displaying the time course of effect-site and plasma concentration, these software packages are wonderful teaching tools, showing, for example, how long a drug takes to achieve its maximal concentration after a bolus. Knowing this delay, called time to peak effect, which is specific for the anesthetic drug and independent of the dose, can help to anticipate the delivery toward the end-point requiring the effect or to synchronize drugs having different time to peak ^9^. From the measured time to peak effect, it is also possible to estimate ke0 for a drug when only a PK but not a full PKPD model is available ^10^.

Software computes the context-sensitive half-time: that is, the delay to decrease by 50% from the current concentration ^11^ or the decrement time (delay to decrease to a chosen concentration such as the concentration expected at recovery) ^9^. When an infusion is terminated, both will depend upon the ratio of the clearance and the rate at which it re-equilibrates between the vascular space and peripheral sites. Because the distribution of anesthetic drugs to peripheral sites almost never reaches steady state and redistribution from the CNS remains a relevant phenomenon during recovery, these decrement times are much faster than the elimination half-life but increase with duration of delivery, especially in drugs with big volumes of distribution and accumulation. When two drugs with different accumulations (for example, propofol + opioid) are administered, decrement time displays may help to adjust the balance to shorten recovery delay ^12^.

But the main clinical use of PK models in anesthesia is undoubtedly target-controlled infusion (TCI). In this delivery mode, the user does not choose the dose to give but directly selects the target plasma or effect-site concentration he or she wants to achieve and maintain ^13^. The software computes by iterations the dose to achieve this “target” as fast as possible without overshoot, drives the pump, gets back the dose which has been given, and updates the infusion rate at regular intervals (1 to 10 sec). If the anesthetist decides to decrease the target concentration, the software stops the pump, continues predicting the concentration at regular intervals, and resumes the infusion as soon as the lower target concentration is reached. In case of interrupted drug delivery (because of an empty syringe or occlusion on the venous line), the software calculates the bolus dose necessary to regain the selected target concentration.

Several manufacturers have implemented TCI software inside high-infusion-rate syringe pumps. They obtained CE mark approval and released a family of devices which today are clinically used worldwide except in North America, where it is still waiting for FDA approval. Released in 1996 for propofol ^14^, TCI devices were later extended to sufentanil, remifentanil, and then alfentanil from 2003.

A faster control of response to incision ^15^ and a better hemodynamic stability have been described with propofol ^16^ or remifentanil ^17^ TCI as well as a good control of spontaneous ventilation ^18, 19^, and use in patient-controlled analgesia mode for postoperative analgesia is possible ^20^. The benefits induced by TCI on the amount of drugs given or on recovery times were heterogeneous and depended widely on the decision criteria chosen in each study to adjust the dosing in both TCI and manual control groups ^21^. Optimizing the combination between bolus and infusion helped reduce the variability of the concentration between patients ^22^. Finally, the most undisputable benefits of TCI were a reduction of workload for the same quality of control ^21^ and better comfort and understanding for anesthetists ^23^.

### Limits of pharmacokinetic models

Despite the clinical usefulness of compartmental models, some limitations have been pointed out over the years.

First, these models do not accurately describe the concentrations during the early phase of administration. The compartmental model assumes that any dose given is instantaneously and homogeneously diluted in the whole blood volume, which is unlikely ^24^. Early distribution needs time and may depend on the location of the venous line and also on cardiac output. This influence may be displayed in models called front-end kinetics, where the volume of the central compartment is expressed by a time-dependent process rather than a constant ^25^. Rarely implemented in drug-delivery software today, these models would provide a better control of the concentration in the first minutes after each delivered dose.

Second, most compartmental models assume that drugs are eliminated only from the central compartment ^24^. If they are also eliminated from tissues such as cisatracurium or remifentanil, this assumption may underestimate elimination clearance and overestimate concentration. Third, the PKPD approach assumes that only the parent drug concentration is responsible for expected effects. When drugs have active metabolite at relevant concentrations, having different PK and different potency, more sophisticated models should be used, as shown with morphine ^26^.

Finally, there is a remaining debate about modeling and TCI in obese patients. Some models used LBM as a covariate ^6, 27^. This LBM is estimated from weight and height by using a historical formula established on moderately obese patients: body mass index (BMI) of less than 42 kg/m ^2^ in men and less than 35 kg/m ^2^ in women. Extrapolating this LBM formula to morbidly obese patients markedly underestimates LBM and results in a relevant underdosage for remifentanil or overdosage for propofol. To avoid this risk, TCI manufacturers have limited BMI to 42 and 35 kg/m ^2^ and recommend compensating for this error by titration. In the future, solutions might use a more extended formula for LBM or choose a model with a scaling value of the real weight for the covariate rather than LBM.

## Pharmacodynamic modeling and drug interactions

Controlling a predicted concentration, as allowed by PKPD modeling and TCI, is not a clinical goal per se. The goal is to control the effects in order to maintain the therapeutic effects while avoiding or minimizing the adverse overdosage effects. Given that the effects are related to the effect-site concentration, which is the fundamental rule for PKPD analysis, the next issue is to understand which concentration induces which effects.

### Therapeutic/surrogate effects and modeling

**Figure 2.**
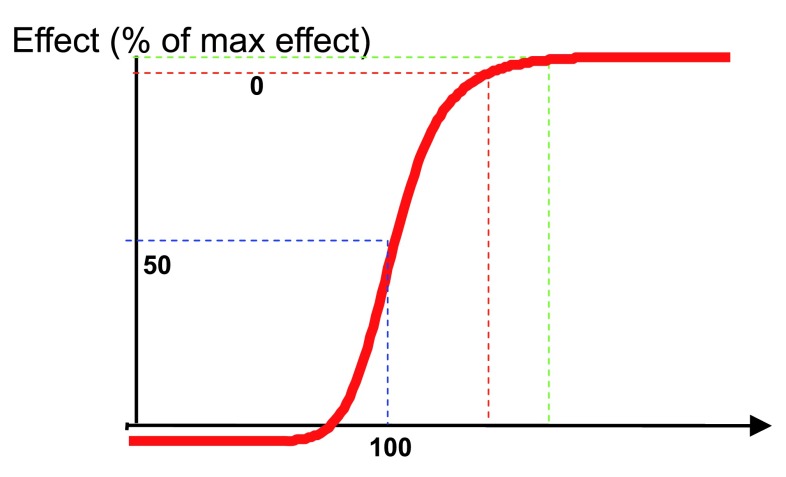
Concentration–effect sigmoidal E _max_ model. General formula: $\text{E}={\text{E}}_{0}+{\text{E}}_{\max}*\frac{{\text{C}}^{\gamma }}{C50^{\gamma }+C^{\gamma }}\text{,}$ inhibitory effect: $\text{E}={\text{E}}_{0}-{\text{E}}_{\max}*\frac{{\text{C}}^{\gamma }}{C50^{\gamma }+C^{\gamma }}\text{,}$ probability (as a percentage): $P=0+100^{*}\frac{{\text{C}}^{\gamma }}{C50^{\gamma }+C^{\gamma }}\text{.}$

As stated in the Introduction, the most-often considered effects in PKPD modeling are quantal effects (yes/no) such as loss of consciousness or absence of response to incision. They are not quantitative but their probability of occurring is. Other therapeutic effects, such as the neuromuscular blockade, are quantitative and measurable. Some adverse effects, such as the degree of respiratory depression, are also quantitative. Finally, some effects are not clinically relevant end-points but surrogate measurable end-points. They are highly correlated with therapeutic effects, i.e. the same concentration of a drug will always induce the same level of both clinical and surrogate effect. Thus, titration of delivery to maintain a “target” chosen value of the surrogate measure will also induce the linked value of the clinical effect. Examples are EEG-related parameters such as Bispectral Index (BIS™), which are often used to titrate anesthesia depth or to express anesthesia depth in concentration-effect modeling ^28^. In all cases, the intensity (or the probability) of the effect increases with the concentration and achieves a maximal value for high concentrations ^29^. Therefore, the relationship between effect-site concentration and each effect can be modeled in most cases by a sigmoidal model described by Hill more than 100 years ago for other drugs ( Figure 2) ^30^.

Each effect will have different Hill curve parameters (C _50_ and γ). For example, propofol C _50_ values have been estimated at 4 µg/ml for loss of consciousness, 10 µg/ml for skin incision, and 17 µg/ml for intubation ^31^.

In clinical practice, the goal is usually not the C _50_ for therapeutic effects because half of the patients would react at that level; instead, the goal is around C _95_ (and the probability of no response is around 95%). A higher probability would require a much higher concentration in this very flat part of the Hill curve and would induce a risk of adverse effects and recovery delay. Conversely, the aim is around C _50_ for EEG effects because the depth of anesthesia inducing maximal EEG effects (flat EEG) requires much higher concentrations than those necessary in clinical practice and the EEG-derived parameters have been designed to offer the maximal sensitivity (steeper part of the Hill curve) in the clinical utility range of concentrations ^32^.

### Drug interactions

To titrate anesthesia to individual needs, the main components of general anesthesia may be split between hypnosis (amnesia and loss of verbal contact), analgesia (limited response to noxious stimulations by movement or autonomic nervous system activation), and muscle relaxation.

Muscle relaxation is fully ensured by neuromuscular blocking agents, and modeling using Hill’s model is simple.

For both other components, no anesthetic drug induces specifically one or the other, but all have a predominant effect on one component and some interaction with other drugs on the other. In other words, opioids (fentanyl, alfentanil, sufentanil, and remifentanil) are mainly analgesics, and huge concentrations induce only a weak sedative effect and moderately enhance the effect of hypnotic drugs on loss of consciousness. Intravenous hypnotics such as propofol, thiopentone, etomidate, or midazolam are mainly hypnotics with almost no analgesic effect when given alone but offer a synergistic interaction with opioids on the analgesia component (that is, the resulting effect is much more intense than the sum of the effects of each drug given alone).

Many studies looked at propofol and opioids ^33, 34^ or volatile anesthetics and opioids ^35^ or three drugs including midazolam ^36^ and described their interactions on hypnosis or analgesia components. They focused on C _50_ and graphically showed the synergism between hypnotic and opioid on a two-dimensional isobole. This interaction may also be modeled by expressing the effect as a linear combination of drug A concentration, drug B concentration, and the product of drug A and drug B concentrations (effect = *α*.[A] + *β*.[B]+ *γ*.[A].[B]), also called the Greco model. The synergism or antagonism is given by the value of *γ* ^37^.

In 2000, Minto and colleagues proposed a response surface approach ^38^. They suggested that any drug combination can be considered a virtual “new” drug of which the effect follows a sigmoidal E _max_ model. The new drug concentration is defined by the ratio of the concentrations of each single drug, each of them weighted by its potency (C _50_).

**Figure 3.**
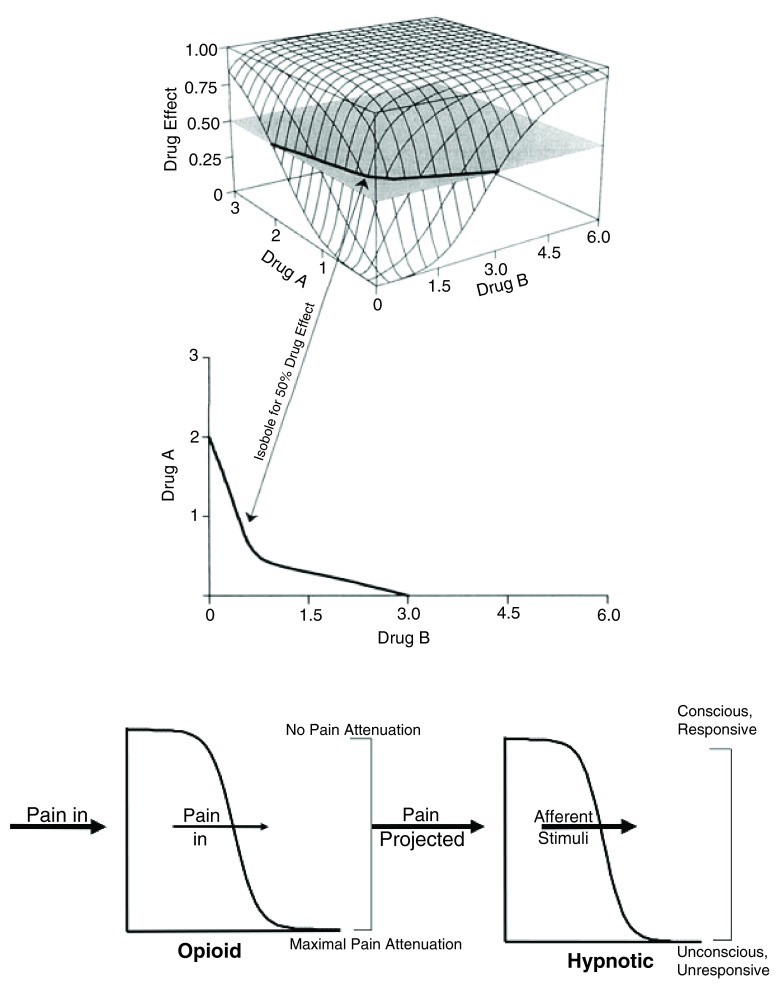
Drug interaction approaches. (Top) Minto response surface. (Bottom) Bouillon indirect model.

The whole model can be displayed on a three-dimensional graph having drug A and B concentrations as x- and y-axis and effect as z-axis ( Figure 3, top). From drug A alone (ratio B/(A+B) = 0) to drug B alone (B/(A+B) = 1), every ratio corresponds to a radial sigmoidal curve. Therefore, the two-dimensional C _50_ isobole described earlier is a particular horizontal slice of the response surface.

Both the Greco and the Minto models assume that the effect is achieved by each single drug. However, in clinical practice, suppression of movement response to surgical stimulation requires high concentrations of propofol whereas loss of consciousness is achieved only at extremely high concentrations of opioid.

Therefore, Bouillon and colleagues proposed another model based on the fact that opioids act not only on the brain but also on ascending neuropathways ^39^. The authors assumed that the control of pain at that level decreases the ascending afferent stimuli going up to the brain and enhances the effect of hypnotics on the brain ( Figure 3, bottom).

This hierarchical model focuses on the reduction of the C _50_ of propofol or volatile anesthetics by opioids to achieve unconsciousness and to suppress response to stimulation but does not assume that opioids on one hand and propofol or volatile anesthetics on the other hand have the same effect. In fact, the hierarchical model by Bouillon and colleagues is a simplification of the Greco model ^40^.

Both models, the empirical one by Minto and hierarchical one by Bouillon express the predicted effect for the whole range of concentrations (not only the concentrations studied), based on a limited number of measures per patient using population analysis ^29^. The hierarchical model seemed to describe the reality more closely, possibly because it is closer to anatomical sites and the mechanism of actions ^41^.

**Figure 4.**
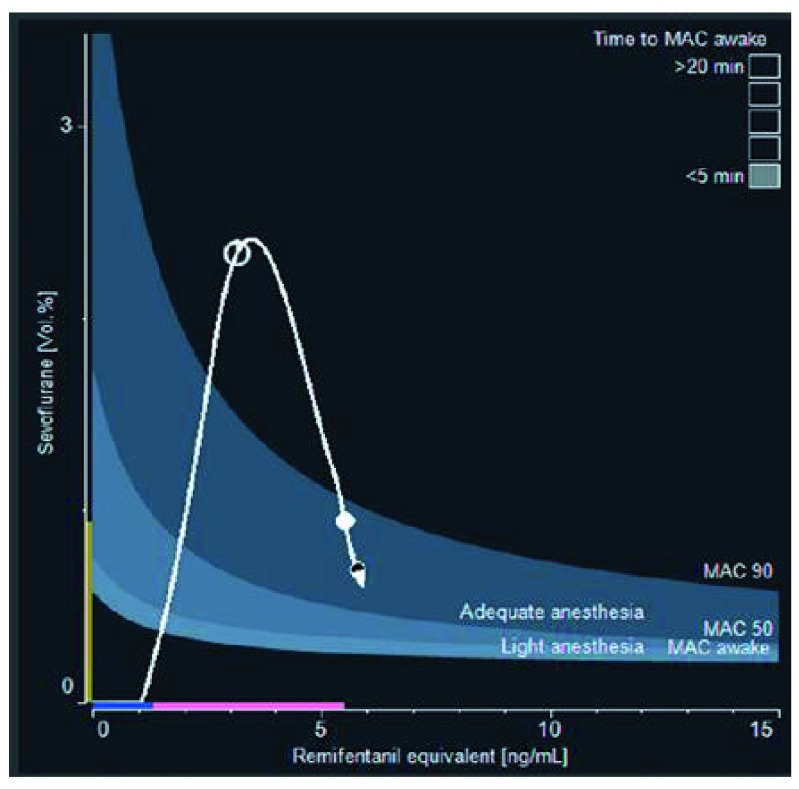
Real-time display of the probability of response to noxious and non-noxious stimuli as a function of hypnotic and opioid predicted concentration. Example of the SmartPilot View™ device (Dräger Medical, Lübeck, Germany). Dark grey area: 50%–90% probability of no motor response to incision. Light grey area: 50%–90% probability of no response to verbal command. White dot: current position of the patient. White arrow: future position in the next 10 minutes without changes in the dosing. White line: past pathway of the patient status since induction. During surgery, this display should complete clinical evaluation: if a patient is responding, it suggests that he or she should be shifted to a deeper anesthesia isobole until the noxious stimulation decreases.

For clinical use, the whole drug interaction model is too difficult to read as a response surface but may be restricted to a two-dimensional family of isoboles showing 50% and 90% probabilities of response to standardized noxious and non-noxious stimuli. The patient’s pharmacological status, based on estimated effect-site opioid and hypnotic drug concentrations, can be displayed in relation to these isoboles in real time as well as in the past and the near future ( Figure 4). This display, designed to help physicians to adjust drug delivery, may be useful to predict the response to noxious stimulations, whereas EEG monitoring predicted better the degree of sedation ^42^.

## Future developments of pharmacokinetic-pharmacodynamic relationship

### Bayesian adjustment

One criticism of TCI is that it is based on the typical population values of the parameters, whereas every patient may differ from this typical value regarding the PK or the PD of the drug, even with models including physiological covariates, or may vary over time from his or her own average PKPD model. If the plasma concentration (or the effect resulting from this concentration) can be measured and compared with the population value, a Bayesian algorithm can adjust the model to make it closer to the patient studied. Initially described with alfentanil and measured concentrations ^43^, this customized PKPD modeling has been applied to rocuronium with repeated measures of effect ^44^. The authors showed that, after two measures, only minor changes in the PK model were observed, suggesting that the intra-individual variability is low compared with inter-individual variability.

In the future, this kind of algorithm may be used in many contexts, such as to adapt propofol PK to online measured concentrations or to real-time EEG effect, especially in patients who are likely very far from the population model (such as those with cardiac failure, obesity, or various organ failures in intensive care).

### Closed loop

Closed loop is often compared to autopilot in airplanes. After a target value for a quantitative effect of anesthetic drugs is defined, a dedicated algorithm repeatedly compares the measured value of this effect to the target and adjusts the drug delivery to achieve and maintain the target. Outside of anesthetic drug delivery, closed loop has been proposed to adjust antihypertensive drugs or controlled ventilation.

In anesthesia, several systems have been developed in the last 30 years, and all of them are still prototypes without a CE mark or FDA approval for clinical use. They drive either muscle relaxants based on neuromuscular blocking agent monitoring or hypnotic delivery based on EEG-derived parameters which have shown a reasonable performance to estimate depth of anesthesia, whereas quantitative monitoring of analgesia and its specificity are still debated.

The simplest algorithm is probably the industry proportional-integral-derivative controller, in which the rate of infusion is adjusted on the basis of the effect error (measured–predicted effect), its time course over time, and its trend. It requires no PKPD model but may oscillate around the target value, is delayed in case of abrupt variations, and may become erratic if no measured value is available because of artifacts ^45^.

Probably safer is the model-based controller, in which the drug is delivered according to a full PKPD model as described above. In this case, the error on the effect does not change the rate of infusion but instead changes the model parameters ^45^, achieving a particular case of Bayesian process. With this approach, if the measure of effect is disturbed after a few measures, the model will be already adapted to minimize inter-individual variability and the accuracy of control will remain sufficient.

Clinically, closed loop has demonstrated an improvement in the duration of control (increasing the duration with desired BIS™ of EEG values from 70% to 90% of the anesthesia time) ^46^ and in the quality of control (also improving blood pressure stability) ^47^. All these clinical results were combined with a marked reduction of the workload ^48^. Many applications can be imagined in both general anesthesia and sedation in the operating room, intensive care, and transport of patients.

## Conclusions

PKPD analysis offers an irreplaceable tool to understand and model the pharmacology of drugs and fits especially well in intravenous anesthetics. From theoretical properties, described using sophisticated mathematical techniques, it has been converted to safe and user-friendly clinical applications in order to provide a more stable but adjustable level of anesthesia. Displaying prediction, it does not replace but rather completes the clinical assessment of the patient. Future perspective will likely pursue this customization of drug delivery.
